# Probing the Na metal solid electrolyte interphase via cryo-transmission electron microscopy

**DOI:** 10.1038/s41467-021-23368-6

**Published:** 2021-05-24

**Authors:** Bing Han, Yucheng Zou, Zhen Zhang, Xuming Yang, Xiaobo Shi, Hong Meng, Hong Wang, Kang Xu, Yonghong Deng, Meng Gu

**Affiliations:** 1 Department of Materials Science and Engineering, Guangdong Provincial Key Laboratory of Energy Materials for Electric Power, Shenzhen Key Laboratory of Solid State Batteries, Southern University of Science and Technology, Shenzhen, China; 2 School of Advanced Materials, Peking University, Shenzhen, China; 3 Battery Science Branch, Sensors and Electron Devices Directorate, US Army Research Laboratory, Adelphi, MD USA

**Keywords:** Batteries, Batteries, Batteries

## Abstract

Cryogenic transmission electron microscopy (cryo-TEM) is a valuable tool recently proposed to investigate battery electrodes. Despite being employed for Li-based battery materials, cryo-TEM measurements for Na-based electrochemical energy storage systems are not commonly reported. In particular, elucidating the chemical and morphological behavior of the Na-metal electrode in contact with a non-aqueous liquid electrolyte solution could provide useful insights that may lead to a better understanding of metal cells during operation. Here, using cryo-TEM, we investigate the effect of fluoroethylene carbonate (FEC) additive on the solid electrolyte interphase (SEI) structure of a Na-metal electrode. Without FEC, the NaPF_6_-containing carbonate-based electrolyte reacts with the metal electrode to produce an unstable SEI, rich in Na_2_CO_3_ and Na_3_PO_4_, which constantly consumes the sodium reservoir of the cell during cycling. When FEC is used, the Na-metal electrode forms a multilayer SEI structure comprising an outer NaF-rich amorphous phase and an inner Na_3_PO_4_ phase. This layered structure stabilizes the SEI and prevents further reactions between the electrolyte and the Na metal.

## Abstract (web summary)

The solid electrolyte interphase (SEI) strongly affects the cycling behaviour of rechargeable alkali metal cells. Here, the authors investigate via cryo-electron microscopy the SEI formed on a Na metal electrode using fluoroethylene carbonate-containing electrolyte.

## Introduction

To avoid the adverse environmental effect caused by burning fossil fuels, we desperately need to explore renewable energy technologies and develop next-generation energy storage systems^1,2^. However, because renewable energy sources such as wind and solar energy are intermittent in their nature, the development of efficient and large-scale energy storage devices capable of storing and balancing their energy output is essential^3,4^. At present, lithium-ion (Li-ion) batteries are the most successful electrochemical energy storage devices, but the scarcity and associated high cost of Li are problematic for large-scale energy storage^5–11^. In contrast, sodium ion (Na-ion) batteries have abundantly available components, high capacity, and low cost, which makes them a very promising alternative for renewable energy storage. A Na-metal anode has a theoretical specific capacity of 1165 mAh g^–1^ and a low electrochemical potential of −2.714 V vs. the standard hydrogen electrode^12–17^. Unfortunately, like Li-metal batteries, Na-metal batteries can fail catastrophically during plating/stripping cycles due to the formation of dendrites and electronically inactive (i.e., “dead”) Na metal^16,18^. In addition, unstable solid electrolyte interphase (SEI) growth caused by continuous reactions between Na metal and electrolyte leads to consumptions of Na source and electrolyte, resulting in low Coulombic efficiency. Due to the higher reactivity of Na metal, the chemical reactions involved in the SEI formation are often more severe than for less reactive Li metal^18,19^. The use of electrolyte additives, such as fluorinated ethylene carbonate (FEC), is reported to improve the cycling performance of Na-based cells significantly^20,21^. However, achieving atomic-resolution characterization of Na-based cells with conventional TEM at room temperature is very challenging because SEI is highly reactive to moisture, air, and electron beams. This reactivity can result in artifacts caused by alkali-metal and air interaction during the sample transfer process^22^, or phase transformations induced by the high-flux of electron beam during conventional TEM examination^23,24^.

Cryo-sample transfer and cryogenic transmission electron microscopy (cryo-TEM) can maintain the native state of sensitive materials and interphases in the batteries at liquid nitrogen temperature for high-resolution imaging^24–27^. For example, Wang et al. reports cryo-TEM and cryogenic electron energy loss spectroscopy (cryo-EELS) measurements to probe the light-elements, intermediates, and bonding in the SEI as a function of battery operation conditions at the atomic scale^22^. The Kourkoutis group has successfully utilized cryo-EELS to discover a novel LiH phase in the Li dendrite, which may be the limiting bottleneck for reversible Li-metal battery cycling^25^. A similar bottleneck has yet to be identified for Na-metal cells, so it is becoming increasingly essential to better understand the atomic-scale chemical mechanisms by which FEC additive seems to improve Na-metal cell cycling performance.

In this work, we deposit Na metal on a TEM grid embedded on the top of a Cu foil (in a Na metal || Cu cell configuration) to probe the SEI formed on the plated Na metal via low-dose cryo-TEM and cryo-EELS. By comparing the respective SEIs formed with and without FEC additive in an electrolyte solution made of ethylene carbonate (EC) and dimethyl carbonate (DMC) solvents and NaPF_6_ salt, we were able to accurately discover that the FEC produces an organized, multilayer SEI with an NaF surficial layer. In doing so, we provide useful insights for designing better Na-metal batteries.

## Results

### Microstructure of the SEI in FEC-free EC:DMC-based electrolyte

**Fig. 1:**
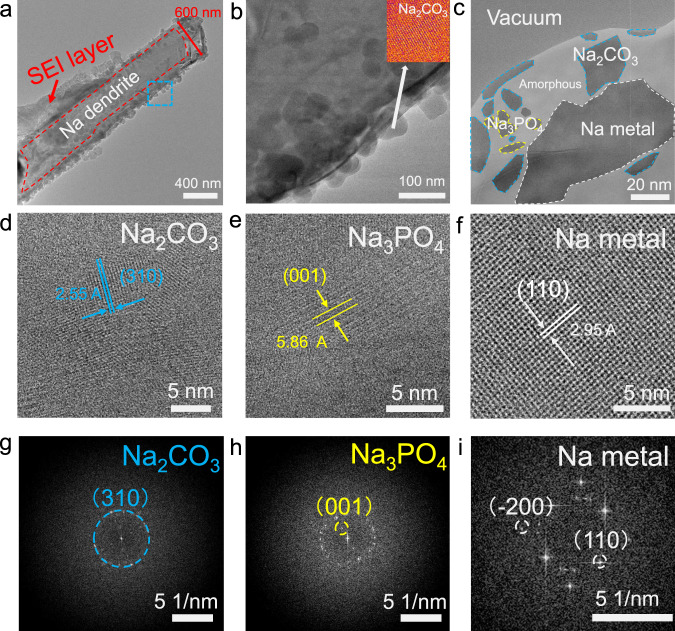
Microstructure of Na-metal dendrite and SEI formed in FEC-free EC:DMC-based electrolyte at the first cycle. **a** Cryo-TEM of the Na dendrite at a low magnification. **b** Magnified TEM of the region outlined in blue in **a**, with inset showing the HRTEM of the spherical Na_2_CO_3_ particles. **c** HRTEM showing the distribution of the SEI components. **d**, **g** Representative HRTEM and FFT of Na_2_CO_3_, **e**, **h**) Na_3_PO_4_, and **f**, **i** Na metal observed in the SEI. (The HRTEMs were obtained with an electron dosage of ~120 e Å^−2^).

Using the FEC-free EC:DMC-based electrolyte, we cycled and then dismantled an asymmetric Na metal || Cu coin cell in an Ar-filled glovebox to carry out postmortem ex situ cryo-TEM analysis after one cycle, and after ten cycles. In the FEC-free EC:DMC-based electrolyte, even in the first electrochemical deposition, the SEI forms on the Na metal with widely varying thicknesses, reaching a few hundred nanometers at the widest points (Fig. 1a). The Na-dendrites grow to approximately 600 nm in diameter, with spherical particulates on their surfaces consisting of mostly crystalline sodium carbonate (Na_2_CO_3_) (Fig. 1b), which may come from the electrolyte solvent reduction. However, some particulates are amorphous, with no crystalline lattices observed. Using a combination of high-resolution TEM (HRTEM) and fast Fourier transform (FFT), we identified specific crystalline components in the SEI. An enlarged view of the SEI film (Fig. 1c) reveals a large piece of Na metal (~100 nm) embedded in the amorphous phase. Apparently, this kind of embedded Na-metal particle is electrically disconnected from the bulk Na anode and is commonly called “dead sodium”, because it is no longer active in the electrochemical cycling. The randomly distributed Na_2_CO_3_, Na_3_PO_4_, and amorphous phase form typical mosaic-type SEI. The regions outlined in blue, yellow, and white in Fig. 1c are identified via HRTEM in Fig. 1d–i as monoclinic Na_2_CO_3_^28^ (space group-*C12/m1*), monoclinic Na_3_PO_4_^29^(space group-*Pm*), and body-centered cubic Na metal^30^ (space group-*Im*3*m*).

**Fig. 2:**
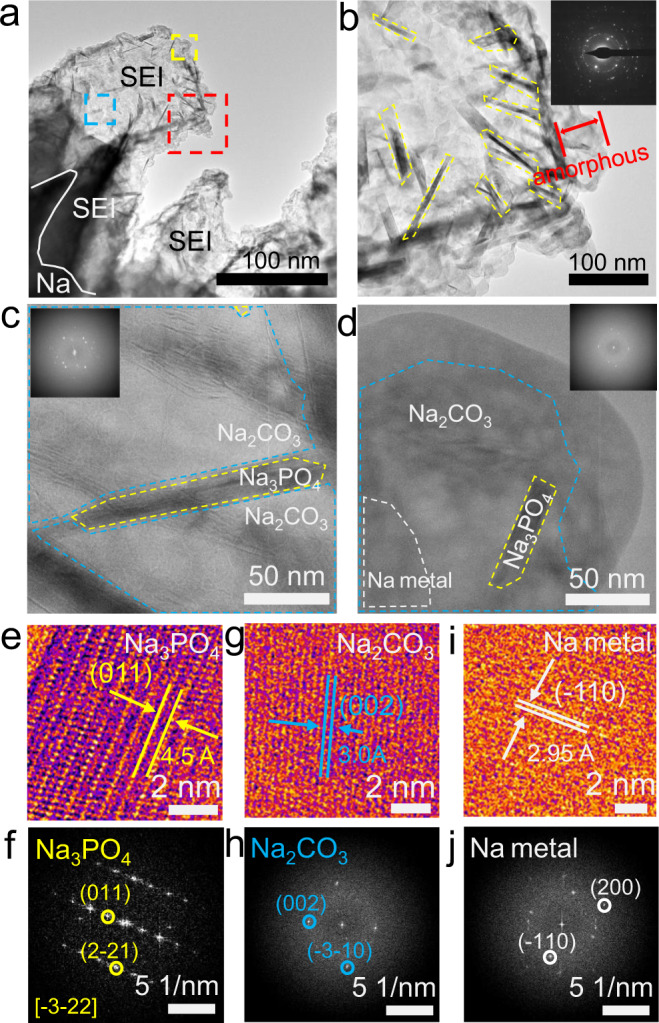
Microstructure of the Na dendrites and SEI formed in FEC-free EC:DMC-based electrolyte after the 10th cycle. **a** Low-magnification cryo-TEM of the thick SEI on Na metal. **b** Magnified view of the regions in **a** outlined in red, **c** blue, and **d** yellow. The inserted FFTs in **c**, **d** show polycrystalline patterns containing Na_3_PO_4_ and Na_2_CO_3_. **e**, **f** Representative HRTEMs and corresponding FFTs of Na_3_PO_4_, **g**, **h** Na_2_CO_3_, and **i**, **j** Na metal. (HRTEMs were obtained with an electron dosage of ~120 e Å^−2^).

After the 10th electrochemical cycling, a thinner region of the SEI was captured (Fig. 2a), where the outermost organic layer is up to ~50 nm at some regions. Many needle-shaped objects are observed outlined in yellow in Fig. 2b, which is distributed randomly inside the SEI. The inserted selected area diffraction pattern in Fig. 2b confirms the polycrystalline nature of the SEI. We identified the different regions in the phase distribution maps (Fig. 2c, d) by monitoring changes in the HRTEM and FFT data (Fig. 2e–j), which show that large pieces of crystalline Na_2_CO_3_ seem to be the dominant phase in the SEI, that the randomly-distributed, needle-shaped objects in Fig. 2b are actually single-crystalline Na_3_PO_4_ rods, and that dead sodium is mingling with amorphous phase and Na_2_CO_3_ in Fig. 2d. The energy dispersive spectroscopy elemental maps of the Na dendrites and SEI after the 10th cycle are displayed in Supplementary Fig. 1.

### Microstructure of the SEI in EC:DMC-FEC electrolyte

**Fig. 3:**
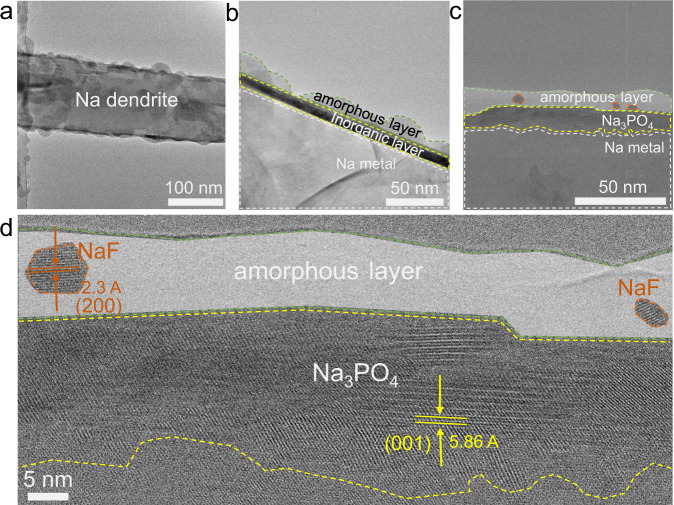
Cryo-TEM analysis of the SEI formed in EC:DMC-FEC electrolyte after the 1st cycle. **a** Low-magnification cryo-TEM image of the Na dendrite. **b** Magnified view of the surface region. **c**, **d** cryo-HRTEM of the SEI showing the distribution of Na metal, NaF outlined in orange, Na_3_PO_4_ outlined in yellow, and amorphous layer highlighted in white.

The addition of FEC to the EC:DMC electrolyte results in dramatic changes to the surface morphology of the Na anode. The low-magnification cryo-TEM image of Na-metal dendrites during the first electrochemical deposition displays an SEI thickness of ~30 nm (Fig. 3a, b), which is much thinner than the SEI formed in the FEC-free EC:DMC-based electrolyte (Fig. 1a). Using a general dual-layer feature of the bright field TEM, we note that the SEI is divided into a light amorphous outer layer and a dark crystalline inorganic inner layer. Inside the amorphous surface layer, we find some randomly distributed NaF islands, as circled in orange in Fig. 3d. HRTEM imaging reveals that the dark inner layer is comprised of clear lattices of Na_3_PO_4_ resting on top of the Na metal (Fig. 3c, d). The Na_3_PO_4_ is polycrystalline and the bottom edge shows many distortions, possibly caused by lattice mismatch with underlying Na metal, and/or a likely off-stoichiometry due to the non-equilibrium electrochemical condition.

**Fig. 4:**
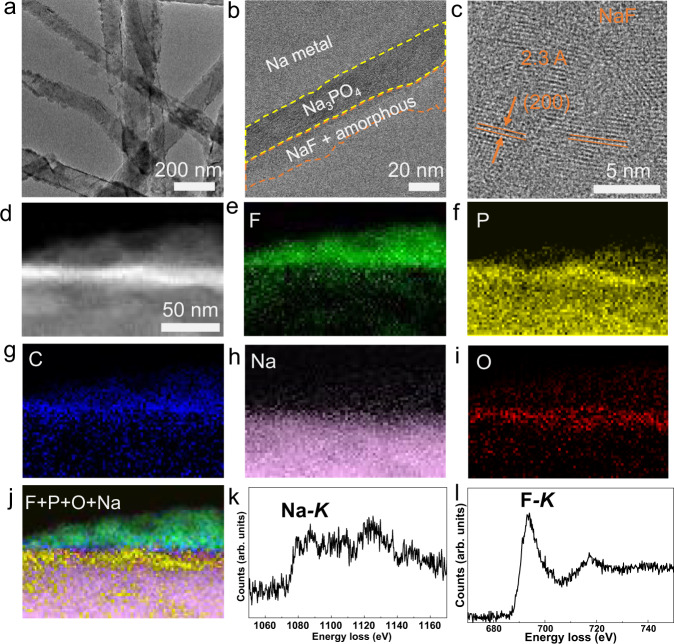
Microstructure and composition analysis of the SEI in EC:DMC-FEC electrolyte after the 10th cycle. **a** Low magnification cryo-TEM image of the Na dendrite. **b** Higher-magnification view of the SEI. **c** HRTEM of surface region containing nanocrystalline NaF. **d** STEM image of SEI layer. EELS elemental maps of **e** F, **f** P, **g** C, **h** Na, **i** O, and **j** composite map; **k**, **l** Na and F *K*-edge EELS spectra.

After the 10th electrochemical cycle, cryo-TEM shows the Na dendrites (Fig. 4a), which are morphologically similar to the dendrites observed in the 1st cycle (Fig. 3a). Higher-magnification cryo-TEM imaging shows a multilayer type morphology with NaF and amorphous layer on top of the inner Na_3_PO_4_ layer (Fig. 4b). The HRTEM image of the top surface in Fig. 4c identifies the (200) planes of NaF clearly. In addition, cryo-scanning transmission electron microscopy (cryo-STEM) and cryo-EELS elemental maps reveal significant F signal in the top surface (Fig. 4d–l). The P signal is localized to the inner regions, indicating that the NaF-rich layer rests on top of the Na_3_PO_4_. The strong C signal in the top surface verifies the composite nature of top surface, including amorphous phase mixing with NaF. The EELS spectra of Na-*K* and F-*K* edges (Fig. 4k, l) also confirm the presence of NaF in the top SEI layer, which is consistent with the HRTEM analysis in Fig. 4c. Please note that because the Na-*K* edge resides at energy range beyond 1000 eV, its signal is weaker than that of other elements.

### XPS analysis of the SEI in EC:DMC-FEC electrolyte

**Fig. 5:**
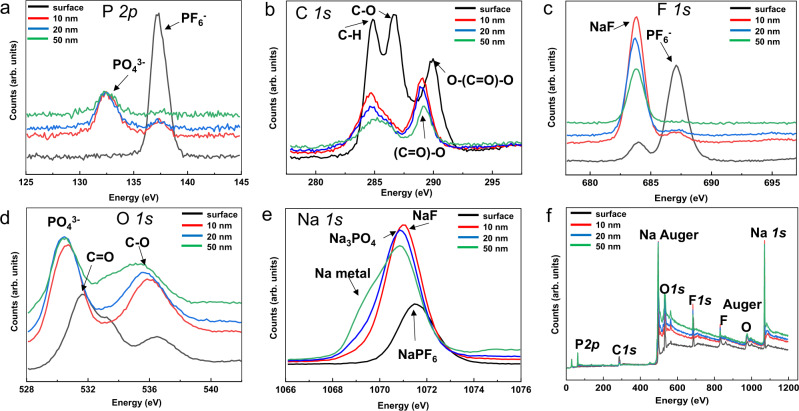
XPS depth profiling analysis of the Na dendrites in EC:DMC-FEC electrolyte at the 1st cycle. **a** P 2*p*, **b** C 1*s*, **c** F 1*s*, **d** O 1*s*, **e** Na 1*s* peaks, **f** full spectra showing all detected elemental peaks.

To validate the cryo-TEM measurement findings and investigate the elemental and bonding compositions of the Na metal depositions obtained at the 1st cycle using EC:DMC-FEC electrolyte, we carried out depth profiling X-ray photoelectron spectroscopy (XPS) measurements at the etching depth of 0 nm (original surface), 10 nm, 20 nm and 50 nm, respectively. Figure 5a present the phosphorus *2p* peaks at different etching depths. According to our analysis, the signal of phosphorus on the sample surface mainly comes from hexafluorophosphate (PF_6_^–^) ions. After etching, the sodium phosphate signal dominates^31^. Figure 5b shows carbon 1*s* peaks at different etching depths, where C 1*s* peaks at the top surface are very different from the inner layers. Obviously, the amorphous phase in the top surface contains more C–H, C–O, and O–(C=O)–O groups than the inner regions^32^. The strong PF_6_^–^ signal in Fig. 5c mainly result from the electrolyte salt residue on the outer surface. After etching, the F 1*s* peaks in Fig. 5c reflects signature of NaF^32^, which is consistent with our cryo-TEM observation of the topmost NaF/amorphous phase composite layer in the SEI. The O 1*s* peaks in Fig. 5d agrees perfectly with the observed multilayer structure using cryo-TEM, where the amorphous phase in the top layer contains mostly C=O and C–O, while the inner layer shows very strong PO_4_^3−^ signal.^32,33^ The Na 1*s* depth profile in Fig. 5e shows the clearly NaPF_6_, NaF, Na_3_PO_4_, and then Na metal stacking sequence in the dendrite, which is consistent with the cryo-TEM. Figure 5f lists the overall XPS peaks of all elements at different etching depths.

Using nuclear magnetic resonance (NMR) spectroscopy, previous research works report that the addition of FEC induces cross-linked polymers similar to polyethylene oxide (PEO) in the SEI^34,35^. Because the resilience and adhesion of the SEI to the Na-metal anode play a critical role in the lifespan of the battery^35^, we compared the XPS and EELS data acquired from sampling the SEI surface to probe the possible composition of the amorphous phases at the 1st and 10th cycle. Based on the XPS and EELS analysis (Supplementary Figs. 2 and 3), the amorphous component in the SEI in both the FEC-free EC:DMC and the EC:DMC-FEC electrolytes contained similar C–O, O–(C=O)–O and C–H functional groups. However, the sample using EC:DMC-based electrolyte contains larger amounts of carbonate groups (–O–(C=O)–O–) than the EC:DMC-FEC sample, possibly due to the large amounts of Na_2_CO_3_ formation in the SEI.

### SEI formation mechanisms

**Fig. 6:**
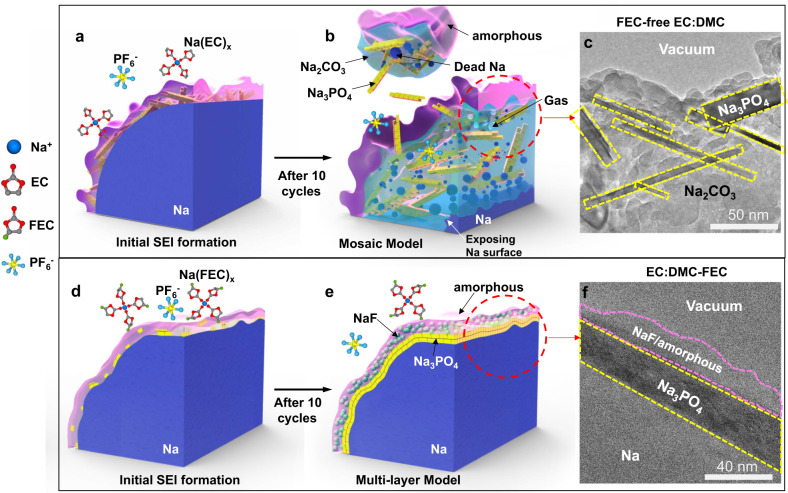
Representation of the SEIs formation during cycling. (**a**) Schematic drawings of the initial SEI nucleation at the 1st cycle and final structure at the 10th cycle, **b** and corresponding cryo-TEM image after ten cycles in the FEC free EC:DMC electrolyte (**c**). Schematic drawings of the initial SEI nucleation at the 1st cycle (**d**) and final structure at the 10th cycle (**e**) and corresponding cryo-TEM image after ten cycles in the EC:DMC-FEC electrolyte (**f**).

Based on our experimental results, we propose the SEI formation mechanisms schematically illustrated in Fig. 6. Because the electron affinity of cyclic carbonates (e.g., EC) is higher than the electron affinity of linear carbonates (e.g., DMC), on a Na metal surface, EC is favorably reduced in an EC:DMC mixed electrolyte^36–39^. At the initial stage of SEI formation in Fig. 6a, the Na^+^ ions solvated by the EC molecules promote a single-electron reduction of the EC, resulting in sodium ethylene decarbonate NaO_2_CO–C_2_H_4_–OCO_2_Na (NEDC), which accumulates and transforms into the amorphous organic/polymer layer identified by cryo-TEM in Figs. 2b and 3c. Because the NEDC has poor hydrolytic stability, it further reacts to form Na_2_CO_3_, ethylene, and CO_2_ in presence of the trace water or acid that always exists in the electrolyte, destabilizing the SEI^40,41^. During battery cycling, the Na_2_CO_3_ on the surface of the SEI further decomposes to generate CO_2_ gas, destroying the intact SEI layer. As the SEI is destroyed, parts of the Na metal are directly exposed to the electrolyte and react to form Na_2_CO_3_. Lucht et al.^42^ reported that the reaction between the different lithium carbonates and the LiPF_6_ salt will yield F_2_PO_2_Li and Li_x_PF_y_O_z._ We infer that the formation of Na_3_PO_4_ is due to similar reactions between sodium carbonates and NaPF_6_ salt in the electrolyte. Further, because of the large CO_2_ production that occurs during NEDC decomposition^40^, the as-formed Na_3_PO_4_ is constantly being disrupted and randomly distributed inside the SEI. Consequently, during stripping, the sodium dendrite may become electrically isolated from the substrate due to nonuniform dissolution rates at different sites of the dendrite, increasing the thickness of the SEI layer and depleting the Na metal. The final SEI formation is a mosaic of NEDC polymer, trapped Na metal, needle-shaped Na_3_PO_4_, and large pieces of Na_2_CO_3_ (Fig. 6b, c).

In sharp contrast, cryo-TEM reveals that the EC:DMC-FEC creates a much thinner SEI, because the FEC reacts with Na metal to form NaF^43^ in a uniform film above the surface of Na_3_PO_4_ at the initial stage of SEI formation in Fig. 6d. The Na_3_PO_4_ and NaF layers efficiently inhibit the reduction of electrolyte, resulting in a sharp decrease of NEDC formation and a commensurate decrease in CO_2_ gas production. The NEDC and Na_2_CO_3_ not covered by the shielding layers quickly decomposes, so the resulting SEI is formed as very dense inorganic Na_3_PO_4_ and NaF/amorphous composite bilayer structure (Fig. 6e, f). The functions of the amorphous organic/polymer phases in the SEI are very important, because they protect the SEI from dramatic volume expansion and shrinkage during cycling^35^, which is strongly correlated with shortened battery life.

### Electrochemical energy storage and impedance spectroscopy measurements

**Fig. 7:**
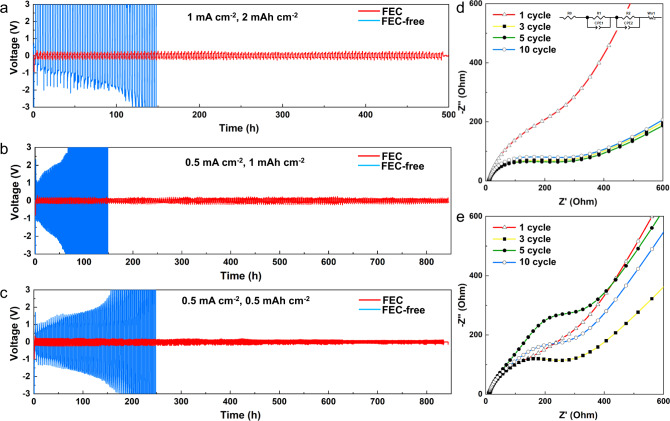
Cell performance comparisons. **a** Electrochemical performance and EIS plot of the symmetric Na metal cells with and without the FEC additive at a current density of 2 mAh cm^−2^; **b** 1 mAh cm^−2^; and **c** 0.5 mAh cm^−2^. EIS plot of the cells using **d** EC:DMC-FEC and **e** FEC-free EC:DMC-based electrolyte at the 1st, 3rd, 5th, 10th cycle.

The correlation between the structural integrity of the SEI and the performance of symmetric Na-metal cells is shown in Fig. 7. The cycling behavior in Na||Na cells with current densities of 0.5, 1, and 2 mA cm^−2^ differed significantly between FEC-free EC:DMC and EC:DMC-FEC electrolytes. Cells containing EC:DMC-FEC electrolyte solutions were able to maintain stable cycling up to 500–800 h at all current densities (Fig. 7a–c), whereas the voltage polarization in the FEC-free EC:DMC-based electrolyte resulted in rapid deterioration after only 50 cycles at the smallest current density (0.5 mA cm^−2^), and instant catastrophic cell failure at a large current density (2 mA cm^−2^). Furthermore, we demonstrate that EC:DMC-FEC electrolyte generates a very consistent impedance and stable SEI layer after the first cycle, while the FEC-free EC:DMC-based electrolyte induced a dynamically changing SEI structure that resulted in increased impedance (Fig. 7d, e) When we fit the impedance of the EC:DMC-FEC cells with an equivalent circuit, the semicircle yielded a sum resistance of 430 Ω in the 1st cycle, 170 Ω in the 3rd cycle, 190 Ω in the 5th cycle, and 200 Ω in the 10th cycle (Supplementary Table 1). In the cells with the FEC-free EC:DMC-based electrolyte, the semicircle yields a resistance of 300 Ω in the 1st cycle, 320 Ω in the 3rd cycle, 680 Ω in the 5th cycle, and 470 Ω in the 10th cycle (Supplementary Table 1). We then assembled and tested a Na metal || Na_3_V_2_O_2_(PO_4_)_2_F (NVOPF) full cell using the FEC-free EC:DMC-based and EC:DMC-FEC electrolyte. Cells with the EC:DMC-FEC electrolyte show excellent cycling stability, while the cells with FEC-free EC:DMC-based electrolyte show significant performance failure after the 5th cycle (Supplementary Fig. 4).

## Discussion

In a cell with FEC-free EC:DMC-based electrolyte, Na metal reacts with electrolyte continuously, depleting both the Na metal and the electrolyte, which causes catastrophic cell failure. Our observations are consistent with previous reports about the effect of FEC in Na-ion cells. Ji et al. reports that FEC addition leads to a thin and uniform SEI layer on the SnSb‐carbon anode for a Na-ion cell^21,44^. Similarly, Fondard et al. proposed that the addition of FEC leads to a decrease in SEI thickness, a reduction in Na_2_CO_3_ formation, and an increase in NaF formation^32^. Researchers calculated the detailed NaF formation route from the reduction of FEC by Na metal using the Monte Carlo/molecular dynamics method^43,45,46^ to show that the NaF formation in the top surface of the SEI can, indeed, stabilize the SEI and limit the solubility issues of the amorphous NEDC compound.

Here, we show the role of the FEC electrolyte additive in tuning the microstructure of SEI on Na metal. When the FEC-free EC:DMC-based electrolyte is used, both Na_2_CO_3_ and Na metal react with the electrolyte, forming a constantly evolving, dynamic SEI. The dead Na metal can be trapped inside the thick SEI, reducing the limited sodium source in the cell and posing potential safety hazards. With the addition of FEC, the FEC-Na metal reaction produces dense and continuous layers of Na_3_PO_4_ and NaF, which cover the Na metal and prevent reactions between the Na metal and electrolyte. Therefore, FEC additive can greatly improve the cell’s Coulombic efficiency and cycling life. This work highlights the use of low-dosage cryo-TEM to directly image the SEI and Na dendrite at the atomic scale, and explain the functional origin of FEC additives for enhanced cell performance. In doing so, it also provides useful insights into the impact of electrolyte additives on the stability of Na-metal cells.

## Methods

### Microscopy and spectroscopy measurements

We use an aberration-corrected FEI Krios G3i microscope to characterize our samples with a Gatan Continuum (1069) EELS spectrometer and Falcon 3 Direct Detection Device. The automatic liquid nitrogen perfusion system can automatically maintain the low temperature of the sample chamber and the lens barrel for days, ensuring high imaging stability. The information limit of the microscope can reach ~0.14 nm. All of the battery samples are dismantled and instantly frozen inside a modified Ar-filled glove box and cryo-transferred to the cryo-TEM. All high-resolution cryo-TEM images are acquired with an electron dosage of ~120 e Å^−2^. The STEM images are acquired at 200 e• nm^−^^2^; the cryo-EELS are acquired using 11 pA current with 0.1 s dwell time at each pixel for core edges. XPS was performed with PHI 5000 VersaProbe-Ulvac-PHI. Each of the Na-metal anodes was placed on a holder and measured with Monochromator Al *K*_α_ (1486.6 eV) X-rays. The spot size of the XPS is around ~500 µm in diameter in this work. The spectra are calibrated using the C 1*s* peak. To avoid electrode contaminations and side reactions, all Na-metal anode samples were transported from the Ar-filled glove box to the XPS chamber in a sealed container that is protected by Ar gas.

### Cells assembly and testing

The EC:DMC-FEC electrolyte used in the experiment contains 1.0 mol/L NaPF_6_ (99% purity, Capchem Co. Ltd., China) dissolved in a solution made of fluoroethylene carbonate (FEC) (99% purity, Capchem Co. Ltd., China):ethylene carbonate (EC) (99% purity, Capchem Co. Ltd., China):dimethyl carbonate (DMC) (99% purity, Kermel, China) with a 10:20:70 volume ratio. In contrast, the FEC-free electrolyte contains only EC and DMC with a 30:70 volume ratio. The water content of the electrolyte is ~7 ppm measured by the Karl Fischer Titrator equipment (Aquatest 2010, USA). Using coin cell type 2025, we assemble the cells with 600 μm-thick and 16 mm-diameter Na-metal foil (99.99% purity, TED PELLA, INC., USA) as the anode. We use 16 mm copper foil (99.99% purity, 10 μm thickness, Kermel, China) as the counter electrode. During the assembly, we place the TEM copper mesh grid (TED PELLA, INC., USA) in the center of the copper foil to collect the deposited Na-metal dendrites for TEM observations. The cells (Na metal|Cu foil) are charged and discharged at a current density of 0.5 or 1.0 mA cm^−2^ and a capacity of 0.5, 1.0, 2.0 mAh cm^−2^_,_ respectively. The charge voltage for the cells is set to 1.0 V vs. Na^+^/Na. The impedance spectroscopy of all cells is carried out using a Solartron analytical electrochemical workstation (mode 1470E) at a frequency range of 1 MHz–0.01 Hz.

The 2025-type symmetric Na||Na coin cells were assembled for electrochemical cycling testing. To be specific, Na foils with a thickness about 200 μm were used as both anode and cathode, nickel foam as spacer, and glass fiber (Whatman) as separator. The electrolyte is 1 M NaPF_6_ in EC:DMC (volume ratio of 30:70) in the FEC-free cell, while the EC:DMC-FEC cell contains 1 M NaPF_6_ in FEC/EC/DMC (volume ratio of 10:20:70). The amount of electrolyte for each cell was controlled to be 60 μL. Galvanostatic cell tests were conducted on LANHE battery testing systems. The discharge time is 1 h, the cut-off voltage is 1.0 V vs. Na^+^/Na, and the value of current is set according to target capacity loading.

The full cell test utilizes a cathode containing NVOPF (160 mg), Super P (20 mg), and PVDF (20 mg) (all with 99.9% purity purchased from Capchem Co. Ltd., China), which are added to 1 mL N-Methyl pyrrolidone (99.95%, Capchem Co. Ltd., China) under continuous stirring (400 r min^−1^) conditions for 12 h. The as-obtained slurry is then homogeneously coated on an Al foil with a blade coater (Laurell, USA). The final mass loading of the NVOPF cathode after drying was ~5.0 mg cm^−2^. The voltage windows for the full cells (Na-metal|NVOPF) were set to 2.8–4.2 V.

## Supplementary information

Supplementary Information

## Data Availability

All relevant data are available from the authors with reasonable requests.
