# Supplementary material for: Probing the Na metal solid electrolyte interphase via cryo-transmission electron microscopy
Source: Nat Commun. 2021 May 24;12:3066. doi: 10.1038/s41467-021-23368-6 (PMC8144392; doi:10.1038/s41467-021-23368-6)
Supplement: Supplementary file 1 — Supplementary Information [file 41467_2021_23368_MOESM1_ESM.pdf]

Supplementary Information

Probing the Na metal solid electrolyte interphase via cryo-transmission electron microscopy

Bing Han[1,†], Yucheng Zou,[1, †] Zhen Zhang,[1] Xuming Yang, [1] Xiaobo Shi,[1] Hong Meng,[2] Hong Wang,[1] Kang Xu,[3] Yonghong Deng,[1*] Meng Gu[1*]

[1]Department of Materials Science and Engineering, Southern University of Science and Technology, Shenzhen 518055, China.
[2]School of Advanced Materials, Peking University, Shenzhen 518055, China.
[3]Battery Science Branch, Sensors and Electron Devices Directorate, US Army Research Laboratory, 2800 Powder Mill Rd., Adelphi, Maryland 20783, United States.

† These authors contributed equally to this work.
*Correspondence to: yhdeng08@163.com (Y. Deng); gum@sustech.edu.cn (M. Gu);

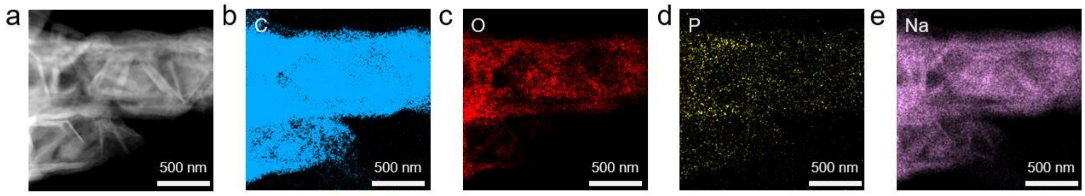

Supplementary Figure 1. a STEM, b. carbon, c. oxygen, d. phosphor, e. sodium EDS map acquired using cryo-TEM on the dendrite cycled with FEC-free EC:DMC-based electrolyte at the 10th cycle.

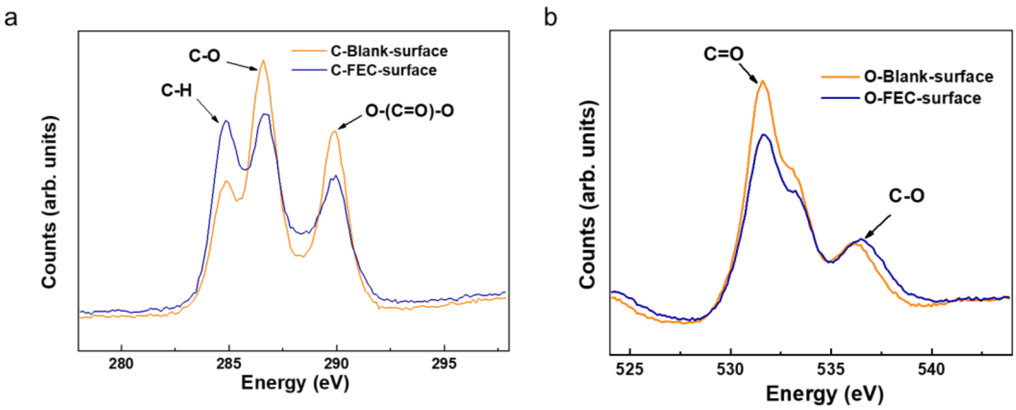

Supplementary Figure 2. XPS comparison of the (**a**) C *1s* and (**b**) O *1s* peaks from plated Na dendrites using FEC-free EC:DMC based electrolyte and EC:DMC-FEC electrolyte at the 1st cycle.

XPS analysis of the amorphous phase in the SEI surface identifies C-H, C-O, and O-(C=O)-O functional groups as shown by Supplementary Figure 2. The signal of C-O and O-(C=O)-O from the Na dendrites cycled in FEC-free electrolyte is much stronger than those sampled in the FEC-containing electrolyte at the 1st cycle.

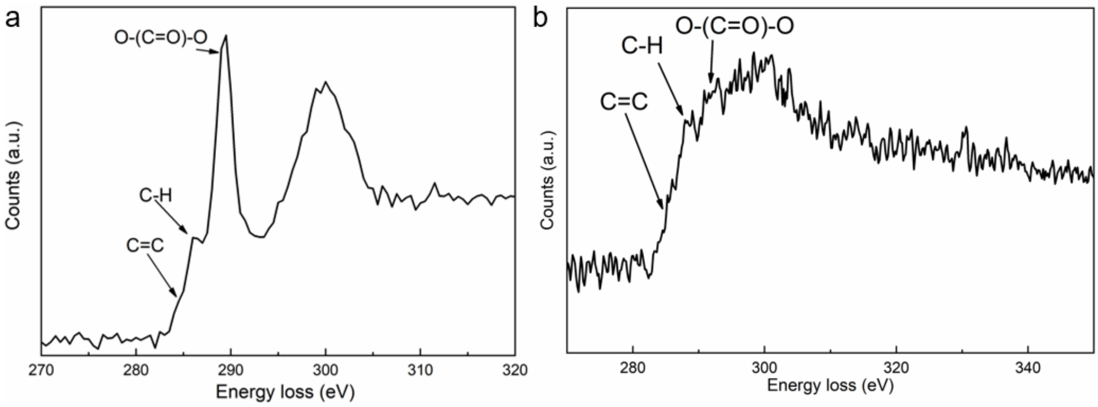

Supplementary Figure 3. Comparison of the C *K* edge EELS spectra of the SEI in two different cells at the 10th cycle. a. FEC-free EC:DMC-based electrolyte. b, with EC:DMC-FEC electrolyte.

EELS analysis in Supplementary Figure 3 from both samples at the 10th cycle proves that the dendrite surface of the FEC-free EC:DMC-based electrolyte sample contains much stronger signal of O-(C=O)-O than the EC:DMC-FEC sample, which may be due to the exposed Na_2CO_3 on the surface.

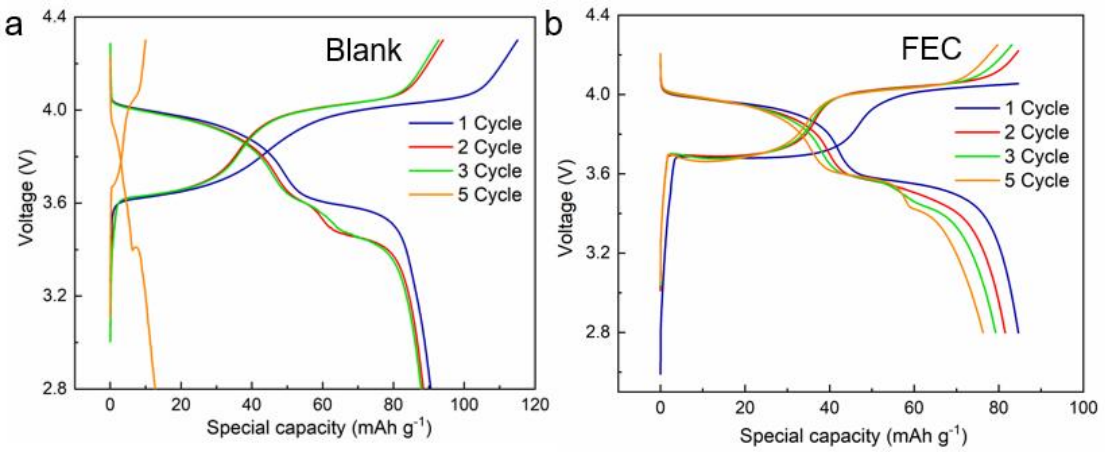

Supplementary Figure 4. The 1st, 2nd,3rd, and 5th charge-discharge curves of NVOPF-sodium metal cells with EC:DMC-FEC and FEC-free EC:DMC-based electrolytes.

Supplementary Figure 4-a&b is the charge-discharge curve of the 1st, 2nd,3rd, and 5th circle of the Na metal-$Na_3V_2O_2(PO_4)_2F$ (NVOPF) full cells using FEC-free EC:DMC-based & EC:DMC-FEC electrolyte, respectively. Comparison of the two graphs clearly shows that cells with FEC additive shows much better cycling stability, while the capacity of the FEC-free cell decreases significantly at the 5th cycle.

Supplementary Table 1. The sum resistance value of each circle of EIS for cells with EC:DMC-FEC electrolyte and FEC-free EC:DMC-based electrolyte.

	First cycle resistance (Ω)	Third cycle resistance (Ω)	Fifth cycle resistance (Ω)	Tenth cycle resistance (Ω)
EC:DMC-FEC	430	170	190	200
EC:DMC	300	320	680	470

By fitting the impedance with an equivalent circuit in Fig. 6, the semicircle yields a resistance of the cells using EC:DMC-FEC electrolyte and FEC-free EC:DMC-based electrolyte. Generally, the cell using EC:DMC-FEC electrolyte has lower resistance than the cell using FEC-free EC:DMC-based electrolyte as cycling increases.
